# Guideline recommendations on the role of the general practitioner in the diagnosis of dementia: a protocol for a scoping review of clinical practice guidelines

**DOI:** 10.12688/hrbopenres.13919.1

**Published:** 2024-07-08

**Authors:** Mary Cronin, Aisling Jennings, Marieke Perry, Irene Hartigan, Séan O'Dowd, Nicola Cornally, Suzanne Timmons, Kieran Walsh, Tony Foley

**Affiliations:** 1 Department of General Practice, School of Medicine, College of Medicine and Health, University College Cork, Cork, Ireland; 2 Department of Geriatrics, Radboud University Medical Centre, Nijmegen, The Netherlands; 3 School of Nursing and Midwifery, University College Cork, Cork, Ireland; 4 HSE National Dementia Office, Tullamore, Ireland; 5 Centre for Gerontology and Rehabilitation, School of Medicine, College of Medicine and Health, University College Cork, Cork, Ireland; 6 School of Pharmacy, University College Cork, Cork, Ireland

**Keywords:** General practice, primary healthcare, practice guidelines, diagnosis, dementia.

## Abstract

**Introduction:**

A timely diagnosis of dementia offers the opportunity of earlier intervention and activation of coordinated care plans. General Practitioners (GPs) play a key role in dementia diagnosis, from symptom recognition to clinical assessment, investigation, diagnosis and onward referral for confirmation of the diagnosis and subtyping. Dementia clinical practice guidelines (CPGs) offer clinicians guidance on dementia care but often do not specifically address the role of the GP in the diagnostic process. This protocol outlines a scoping review to identify evidence-based dementia clinical practice guidelines and map the recommended role of GPs in the diagnosis of dementia.

**Method:**

The scoping review will be conducted using the Arksey and O'Malley framework, and the Preferred Reporting Items for Systematic Reviews and Meta-Analysis extension for scoping reviews (PRISMA-ScR) will be used to guide the reporting. We will search five electronic databases (PubMed, CINAHL, Embase, PsycINFO, Cochrane Library) for dementia CPGs published since 2019. CPGs are often not published in peer-reviewed journals; therefore, a parallel search of relevant grey literature will be conducted. We will also search the websites of GP professional organisations and guideline developers. Two reviewers will independently screen all articles based on inclusion criteria, with conflicts resolved by a third reviewer.

**Conclusion:**

This scoping review will examine up-to-date dementia CPGs to determine recommendations for the role of GPs in the assessment, investigation, diagnosis and onward referral of patients with suspected dementia to secondary care.

## Introduction

Dementia is an acquired cognitive function loss across several domains ^1^ with progressive and potentially severe effects on day-to-day living for patients and their families. Globally, approximately 55 million people live with dementia, with an annual incidence of nearly 10 million cases ^2^, making dementia care a global health priority ^3^. A timely diagnosis of dementia is important as it can improve the quality of life of people with dementia and their family caregivers ^4^. Furthermore, with the emergence of disease-modifying therapies (DMTs), early identification and onward referral for further specialist evaluation is critical as DMTs are indicated for use in early disease stages due to their potential to slow progression but not reverse existing symptoms ^5^.

General Practitioners (GPs), as the usual first point of contact, have a key role in symptom recognition, assessment and referral of patients with suspected dementia. This may involve the direct diagnosis of dementia by the GP or, more usually, the completion of a detailed clinical assessment, relevant investigations and the exclusion of other differential diagnoses prior to onward referral to specialist assessment services for confirmation of the diagnosis and sub-typing ^6– 8^.

GPs often look to evidence-based clinical practice guidelines (CPGs) to facilitate decision-making in the clinical setting ^9^. A recent systematic review of CPGs for dementia found that many publications include information on diagnosis, treatment and monitoring, with a small number of more recent publications also including screening ^10^. However, despite their central position, the specific role of GPs in the diagnostic process has not been examined within CPGs. Recommendations around clinical assessment and onward referral from primary to secondary care are often not addressed or are unclear in CPGs. For example, the National Institute for Health and Care Excellence (NICE) guideline indicates referral to specialist services when dementia is still suspected after reversible causes of cognitive decline or cognitive impairment from medicines have been investigated ^7^. In comparison, the Scottish Intercollegiate Guidelines Network (SIGN) guideline suggests several cognitive tests that may be used to identify patients who would benefit from referral to secondary care ^11^.

To the best of our knowledge, no systematic attempts have been made to compare recommendations from existing dementia CPGs with respect to the role of the GP in the diagnosis of dementia. This review aims to identify CPGs that include a focus on the diagnosis of dementia and examine the specific role of GPs in the clinical assessment, investigation, diagnosis and referral of patients to secondary care. The review findings will inform the development of a dementia diagnosis guideline for GPs in Ireland and a dementia referral form that may be used when referring a patient with suspected cognitive impairment or dementia from primary to secondary care.

## Method

The method will follow the procedure outlined by Arksey and O'Malley ^12^ while incorporating additional revisions and suggestions for enhancing and strengthening the framework ^13, 14^. Therefore, the review follows a six-step process including: (1) identifying the research question, (2) identifying relevant studies, (3) study selection, (4) charting the data, (5) collating, summarising and reporting the results, (6) consultation exercise with stakeholders. Each of the six steps in the framework are outlined in detail below. The Preferred Reporting Items for Systematic Reviews and Meta-Analysis extension for scoping reviews (PRISMA-ScR) ^15^ will guide the reporting of the scoping review. The PRISMA-ScR provides a standardised system for researchers to report scoping reviews and includes twenty essential and two optional items in a checklist format. The checklist includes the key items considered essential for providing full transparency around the research process. The completed checklist will be provided in the scoping review publication. This scoping review protocol was registered with Open Science Framework on 17th June, 2024.

### 1. Identifying the research question

The Population, Concept, Context (PCC) model ^16, 17^, widely recommended for scoping reviews, was used to determine the focus of the research question and to formulate inclusion and exclusion criteria. 

Search terms (See Table 1) were developed by the research team using a literature review and informed by previous systematic and scoping reviews related to clinical practice guidelines and dementia ^10, 18, 19^. An information specialist from University College Cork was consulted to finalise the research terms. The search terms will be refined further based on retrieved abstracts ^13^.

**Table 1.** Search terms.

	Search terms
Patients with cognitive impairment or dementia	dementia* OR Alzheimer* OR “cognitive impairment” OR “Cognitive decline” OR “cognitive dysfunction”
Management or diagnosis of dementia	Diagnos* OR screen* OR “best clinical practice” OR “disease management” OR manage* OR “principles of car*” OR “comprehensive car*”
Guideline	Guid* OR “best practice*” OR procedure* OR “Clinical Practice Guideline*” OR recommend*

Overarching research question: What is the recommended role of the GP in the diagnosis of dementia in CPGs?

Specifically, the review will focus on the following questions:

1. What CPGs on dementia care include a focus on the diagnostic process in the general practice setting?
2. What are the recommendations regarding the role of the GP in the clinical assessment, investigation, and diagnosis of dementia?
3. What is the recommended referral process when referring a patient with suspected dementia from primary to secondary care?

### 2. Identifying relevant studies

Searches will be carried out on databases of peer-reviewed literature (PubMed, CINAHL, Embase, PsycINFO, Cochrane Library). We will use the alerts feature on databases to keep up to date with any new publications throughout the course of the review. Given that many CPGs may not be published in peer-reviewed databases, we will primarily search the grey literature. The grey literature search will include Lenus (the Irish Health Repository), National Institute for Health and Care Excellence (NICE) and Scottish Intercollegiate Guideline Network (SIGN) websites and generic search engines, i.e. Google and Google Scholar (first 200 citations). In addition, we will search appropriate databases for guidelines including, but not limited to, Guideline Central, Guidelines International Network, UpToDate, TRIP, and the Agency for Healthcare Research and Quality, using combinations of the keywords used in the bibliographic database search. We will also search the websites of GP professional organisations both nationally and internationally. Where there is more than one guideline from the same author or organisation we will use the most up-to-date version. We will also search the reference lists of all included CPGs.

Searches will be limited to titles and abstracts, and all identified citations will be uploaded to Covidence for screening and data management purposes.

The literature search will be limited to publications from January 2019 to May 2024. These timelines were selected to reflect the evolution of clinical practice and to update the literature since the publication of the most recent CPG on dementia care for GPs in Ireland in 2019 ^20^.

### 3. Study selection

The title and abstract of papers will be screened initially, and full-text papers will then be reviewed for inclusion. Clinical practice guidelines will be translated into English when required. Full-text studies that do not meet the inclusion criteria will be excluded, and the reasons for exclusion will be provided in an appendix in the final scoping review (See Table 2 for inclusion/exclusion criteria). Where there is conflict, another member of the multidisciplinary research team will act as a third reviewer. This systematic team approach to study selection is recommended to ensure rigour ^13^.

**Table 2.** Eligibility criteria.

*Inclusion criteria*	*Exclusion criteria*
Papers from the past five years (2019-2024) will be included to gain up-to-date clinical guidance. Guidelines for the management or diagnosis of Mild Cognitive Impairment (MCI), early Alzheimer’s Disease and related dementias. Evidence-based guidelines produced by national and international groups to guide clinical practice following formal procedures for practice guideline development. Appropriate for use in primary care. Relevant to the diagnosis of dementia in primary care. Relevance determined by the inclusion of: ● Screening to identify patients with MCI or Dementia ● Clinical assessment ● Diagnosis ● Elimination of other possible causes of symptoms ● Investigation of dementia ● Recommendations for referral Guidelines in any language.	Guidelines for specific populations, e.g. guidelines for dementia in young adults or those with ID. Guidelines not evidence-based or where the guideline development is not transparent. Regional guidelines. Research studies, academic journal articles such as those examining guideline adherence, compliance, development, or evaluation. Editorials. Conference proceedings and theses. Study Protocols. Guidelines specific to genetic testing, risk factors, dementia prevention, post-diagnosis care, palliative care, treatment or therapies for the management of dementia. Guidelines specific for other health professionals, i.e., nurses, Allied Healthcare Professionals, or caregivers. Guidelines for use in hospital settings. National strategies or policies.

### 4. Charting of the data

Data charting involves charting the data according to key issues and themes ^12^. We will also conduct another step to charting, which involves two reviewers independently charting five to ten papers using the form and then consulting to discuss if their approach is consistent ^13^. Therefore, we will develop a draft form to include items such as 1) authors, 2) year of publication, 3) country of origin, 4) aims/ objectives, 5) type of guideline/guidance, 6) clinical history, 7) physical examination 8) cognitive screening tests 9) investigations, 10) exclusion of other diagnosis 11) communication to other healthcare professionals 12) referral to specialist services. We will review a number of papers using the form and will consult and update the form, as necessary. Following the finalisation of the charting form, we will chart the remaining papers and consult to finalise the charting in an iterative process.

### 5. Summarising and reporting results

Two reviewers will summarise findings from the extracted data to include a descriptive numerical summary of the characteristics of the included guidelines. The research team will produce a complete synthesis of recommendations for referral from primary care to secondary care. Results will be reported using the PRISMA-ScR guidelines ^15^. Each research question will be reported separately and presented in a tabular form and as a narrative summary.

### 6. Consultation exercise with stakeholders

Arksey & O’Malley suggested that a consultation exercise with stakeholders is an optional step ^12^. However, Levac *et al.* and Daudt *et al.* suggest that this should be required for a scoping review ^13, 14^. We will engage with GPs, secondary care specialists, multidisciplinary healthcare professionals and with The Dementia Research Advisory Team (DRAT) of the Alzheimer’s Society of Ireland. DRAT is a group of people living with dementia and carers/supporters who are involved in dementia research as co-researchers.

## Conclusions

The findings from this scoping review will inform the development of a dementia diagnosis and referral guideline for GPs and a referral form to support referral from primary care to secondary care for patients with suspected cognitive impairment or dementia. The rigorous and transparent methodology and a strong multidisciplinary team approach will ensure that the guideline and referral form is based on rich data and provides appropriate information to GPs. The research findings will also be submitted for publication in relevant peer-reviewed journals and presented at conferences.

## Data availiability

No data are associated with this article.
