# Supplementary material for: Guideline recommendations on the role of the general practitioner in the diagnosis of dementia: a protocol for a scoping review of clinical practice guidelines
Source: HRB Open Res. 2024 Jul 8;7:46. [Version 1] doi: 10.12688/hrbopenres.13919.1 (PMC11836558; doi:10.12688/hrbopenres.13919.1)
Supplement: Peer review history

# Reviewer report: Reviewer response for version 1

**DOI:** 10.21956/hrbopenres.15268.r43930

**Published:** 2024-12-18

**Authors:** Soo Borson

**Affiliations:** 1 University of Southern California, Los Angeles, USA

This paper describes a protocol for a scoping review of guidelines for GP diagnosis of dementia. While it's questionable whether a protocol paper for a scoping review requires publication on its own - rather than simply as part of the completed review when submitted - this is well done.  A particular strength is the authors' intent to consult a range of relevant stakeholders as part of the scoping process.

 Suggestions:

 1. I would strongly urge the authors to frame the 'why' of GP dementia diagnosis, i.e. how making a dementia diagnosis in primary care informs patient (not just disease) management; what do GPs need to know about the individual and the family in order to provide primary care for people with dementia - regardless of specialist involvement? 

 2. I recommend similar attention to the 'why' of specialist referrals for diagnosis. We know that patterns of diagnosis and management of dementia are heavily dependent on national, local, and specific practice-based opportunities, which are not equitably distributed.  There has been a longterm mismatch between recognition that GPs do most of the diagnosing and managing of patients with dementia and the 'knee-jerk' expectation that all patients with possible cognitive disorders need to see specialists; we know this is both infeasible, invalid when considered from a global perspective, and likely wasteful of scarce specialist resources (no offense to specialists; I am one). It would be most welcome to have a critical, evidence-based review of decision points regarding who, when, and why specialist referral is important and useful. 

 3. There will need to be some attention to another scarce resource - the availability of high-specificity diagnostic testing for etiology of dementia, and of disease-modifying treatments for Alzheimer's disease, which varies with country, region, population, health system, and many other ecological determinants. Identifying candidates for such therapies is a major driver of specialist referral in some sectors of US health care; it would be valuable to consider this from a transnational perspective.

Is the study design appropriate for the research question?

Yes

Is the rationale for, and objectives of, the study clearly described?

Partly

Are sufficient details of the methods provided to allow replication by others?

Yes

Are the datasets clearly presented in a useable and accessible format?

Not applicable

Reviewer Expertise:

Dementia in primary care; early detection of dementia; complex care approaches.

I confirm that I have read this submission and believe that I have an appropriate level of expertise to confirm that it is of an acceptable scientific standard, however I have significant reservations, as outlined above.

# Response

**Published:** 2025-02-11

**Authors:** Mary Cronin

**Affiliations:** Department of General Practice, University College Cork School of Medicine, Cork, County Cork, Ireland

Many thanks for the very helpful, constructive comments around the ‘why’ of GP diagnosis, the ‘why’ of referral to specialists for diagnosis and also the introduction of DMTs and the scarcity of resources. We have added two paragraphs to the Introduction section to address these important points. We feel this has strengthened the paper significantly, adding a real-world context to the diagnostic debate. In the full scoping review, we will further elaborate on these important points

**Point by point response:**

**Comment 1:**

**Reviewer’s Comment**

 “I would strongly urge the authors to frame the ‘why’ of GP dementia diagnosis, i.e. how making a dementia diagnosis in primary care informs patient (not just disease) management; what do GPs need to know about the individual and the family in order to provide primary care for people with dementia – regardless of specialist involvement?”

**RESPONSE**: Thank you for this comment.  We acknowledge that there is a complexity to the ‘why’ of GP dementia diagnosis and how it informs patient care.  We have added the following text to address this point.

*‘While GPs have been criticised for low identification and dementia diagnosis rates in primary care* *(13)* *.Research has found that many GPs take a nuanced, patient-centred approach* *(14)* *. GPs undertake a complex, decision-making process, whereby they balance the risks and benefits of making the diagnosis* *(15)* *.  GPs compare competing patient priorities, when deciding when to initiate the formal diagnostic process and when to refer onwards to secondary care for confirmation of a suspected diagnosis of dementia. Even in the absence of a formal diagnosis or disclosure of a diagnosis of dementia,* *GPs activate a range of dementia care services and supports for their patients* *(14)* *.’(Introduction Paragraph 2).*

**Comment 2:**

**Reviewer’s Comment:**

 “I recommend similar attention to the ‘why’ of specialist referrals for diagnosis. ​ We know that patterns of diagnosis and management of dementia are heavily dependent on national, local, and specific practice-based opportunities, which are not equitably distributed. ​ There has been a long-term mismatch between recognition that GPs do most of the diagnosing and managing of patients with dementia and the ‘knee-jerk’ expectation that all patients with possible cognitive disorders need to see specialists; we know this is both infeasible, invalid when considered from a global perspective, and likely wasteful of scarce specialist resources (no offense to specialists; I am one). ​ It would be most welcome to have a critical, evidence-based review of decision points regarding who, when, and why specialist referral is important and useful.” ​

**RESPONSE: **We appreciate your comment and agree that the rationale for specialist referrals needs to be clearly articulated. We have added the text below and expanded further in response to comment no.3.

*‘When deciding whether to initiate the formal diagnostic and referral process, GPs weigh up many different competing issues* *related to patient autonomy, consent, risks, patient needs, planning for the future, optimising treatments and access to dementia care services* *(14, 15)* *. Upon making a decision to refer to specialist services, dementia diagnostic and care pathways can be difficult to navigate for both GPs and for people living with dementia and their carers,* *(16, 17)* *leading to further diagnostic delays.’ (Introduction Paragraph 3)*

**Comment 3:**

**Reviewer’s Comment:**

 "There will need to be some attention to another scarce resource - the availability of high-specificity diagnostic testing for etiology of dementia, and of disease-modifying treatments for Alzheimer's disease, which varies with country, region, population, health system, and many other ecological determinants. ​ Identifying candidates for such therapies is a major driver of specialist referral in some sectors of US health care; it would be valuable to consider this from a transnational perspective." ​

**RESPONSE:** Thank you for this comment regarding disease modifying therapies and limited resources. We have added the following text to address this point.

*‘Up until now, the absence of effective disease modifying therapies (DMTs) has led to national dementia strategy recommendations for a ‘timely’ diagnosis of dementia as opposed to an ‘early’ diagnosis i.e. at the right time for the patients, taking the risks and benefits of diagnosis into consideration (Vinay). However, the move towards a biological diagnosis of Alzheimer’s disease and the introduction of* *the first anti-amyloid immunotherapy DMTs for the treatment of patients with early symptomatic Alzheimer’s disease will inevitably necessitate a shift towards earlier identification and diagnosis (Dobson).  Healthcare systems are poorly prepared for this paradigm shift in diagnosis and treatment.* *The emergence of DMTs will likely increase the pressures and demands on primary care and on the limited resources of healthcare systems too (Smith).* *​* *It will be important for GPs to identify potentially eligible patients with cognitive disorders, arrange early referral to specialist centres and coordinate care for patients during and after treatment. While the introduction of DMTs brings hope for many, evidence-informed, clear and equitable diagnostic pathways from primary care to specialist services will be urgently needed.’(Introduction paragraph 3)*

# Reviewer report: Reviewer response for version 1

**DOI:** 10.21956/hrbopenres.15268.r42855

**Published:** 2024-11-25

**Authors:** Emer O'Brien

**Affiliations:** 1 Department of General Practice, RCSI, Dublin, Ireland

Thank you for the opportunity to review this scoping review protocol on evidence relating to GP care of patients with dementia.

 Below are some comments for consideration by the authors.

**Overall**

 The rational for the review is clearly outlined. The aim is to map the role of the GP in the diagnosis of dementia. I would suggest it might be sufficient to search International GP professional organisations specifically to determine best practice relating to the role of the GP in this process. This may require modification of the search strategy to include primary care/general practice and a specific focus on searching/contacting GP professional organisations, as these guidelines are not always available through database searches, as outlined by the authors.

 More specifically this review wishes to explore *‘the specific role of the GP in… referral of patients to secondary care.* CPGs may outline recommendations for referral between primary and secondary care but this interface will be different depending on the role of the GP in the healthcare system - the gatekeeper role. Another consideration might be who within the health system develops guidelines for use in general practice. For example, in the UK NICE hold this central function and there may be GP representatives on the guideline working group. In comparison in the Netherlands, it is the role of the GP professional organisation (NHG) to develop guidelines. I would suggest further exploration of these issues would be valuable in the introduction.

**Further considerations**

 The third objective is to determine what is the recommended referral process – I wonder will these details relating to the referral process be included in CPGs, this information may be available via international GP professional organisations.

 In the methods section - it might be useful if the authors provide a definition for the word 'published’ eg. available on GP professional organisations websites (which can include national or international guidelines endorsed by professional organisations). As above and in addition, given the 2023 SR by Monfared et al, it may be sufficient for the authors to review recommendations specific to general practice.

 Identifying relevant studies – ‘ *we will also search the websites of GP professional organisations both nationally and internationally*’ Consideration will need to be given to access – if the guidelines are not open access will these be excluded or will the summary (which often is open access) be included. Will the authors contact the organisation directly to gain access to the required CPGs.

 Inclusion criteria – *appropriate* for use in primary care – if not including general practice/primary care in the search terms, the word appropriate will need to be clearly defined. Exclusion – *guidelines for use in hospital settings* – suggest clarify if hospital setting and secondary care are synonymous

 Guideline/guidance – the inclusion criteria is specific to 'guideline’ therefore suggest remove guidance or include an explanation for the word 'guidance' in the inclusion criteria.

 Consultation – this is a most welcome step as will ensure both healthcare professional and patient involvement which should increase the likelihood of implementation."

Is the study design appropriate for the research question?

Yes

Is the rationale for, and objectives of, the study clearly described?

Partly

Are sufficient details of the methods provided to allow replication by others?

Yes

Are the datasets clearly presented in a useable and accessible format?

Not applicable

Reviewer Expertise:

general practice, scoping review, systematic review, evidence based practice

I confirm that I have read this submission and believe that I have an appropriate level of expertise to confirm that it is of an acceptable scientific standard, however I have significant reservations, as outlined above.

# Response

**Published:** 2025-02-11

**Authors:** Mary Cronin

**Affiliations:** Department of General Practice, University College Cork School of Medicine, Cork, County Cork, Ireland

Many thanks for this helpful review, which raised valid points about the availability and development of Clinical Practice Guidelines and the international context, all of which add value to the protocol paper. We have added information to the introduction and method to address these points, and they will be further explored in the discussion of our findings in the scoping review paper.

**Point-by-point response:**

**Comment 1**

**Reviewer’s comment** **:**

 “The rational for the review is clearly outlined. The aim is to map the role of the GP in the diagnosis of dementia. I would suggest it might be sufficient to search International GP professional organisations specifically to determine best practice relating to the role of the GP in this process. This may require modification of the search strategy to include primary care/general practice and a specific focus on searching/contacting GP professional organisations, as these guidelines are not always available through database searches, as outlined by the authors.”

**RESPONSE**: Thank you for this observation. This was carefully considered during our preliminary searches to refine the search terms. Our initial searches indicated that CPGs for dementia care were published by both national organisations and GP professional organisations. Therefore, to ensure a rigorous approach, we will search the websites of GP professional organisations, both nationally and internationally, as well as the peer-review databases and other sources of grey literature. Where no guidelines are available on the website, we will email the organisations to request copies. We have now amended the protocol method section to reflect this. The following text has been added.

*We will also search the websites of GP professional organisations both nationally and internationally as guidelines are often made available for GPs through these platforms.* *Where dementia guidelines are unavailable (or inaccessible) on the professional organisation websites, we will contact them via email to request a copy. Google Translate will be used to increase the likelihood of a response from international GP organisations.*

**Comment 2**

**Reviewer’s comment:**

 “More specifically, this review wishes to explore *‘the specific role of the GP in… referral of patients to secondary care.* CPGs may outline recommendations for referral between primary and secondary care but this interface will be different depending on the role of the GP in the healthcare system - the gatekeeper role. Another consideration might be who within the health system develops guidelines for use in general practice. For example, in the UK NICE hold this central function and there may be GP representatives on the guideline working group. In comparison in the Netherlands, it is the role of the GP professional organisation (NHG) to develop guidelines. I would suggest further exploration of these issues would be valuable in the introduction.” 

**RESPONSE**: Thank you. In the introduction, we have now mentioned the complexity of differing health systems internationally, which will also be addressed in more detail in the scoping review paper. We have also acknowledged that different organisations may be responsible for guideline development, and similarly, we aim to address this further based on our findings. The following text has been added to the introduction.

*In terms of the dementia diagnostic pathway, GPs play varying roles in healthcare systems worldwide, with a significant emphasis on a gatekeeping role in many countries. In the UK and Dutch healthcare systems, GPs serve as gatekeepers, managing patient access to specialists and ensuring efficient use of resources* *(9, 10)* *. Similarly, in Australia, GPs control patient referrals to specialists and diagnostic services, promoting cost-effectiveness and preventive care* *(11)* *. Conversely, in the United States, GPs have a less centralized gatekeeping role, as patients often have direct access to specialists, depending on insurance plans* *(12)* *. These varying GP roles reflect differing healthcare priorities, from cost containment to patient autonomy.* *(Introduction paragraph 2)*

*The development of CPGs can vary with some countries, such as The Netherlands, developing practice guidelines specifically for GPs. In contrast, in other countries such as the UK, guidelines are developed by national organisations (Introduction paragraph 5)*

**Comment 3**

**Reviewer’s comment:**

 “The third objective is to determine the recommended referral process. I wonder if these details relating to the referral process will be included in CPGs. This information may be available via international GP professional organisations.” 

**RESPONSE: **Thank you; this is a very valid point. However, this review examines Clinical Practice Guidelines (CPGs) to establish the recommended role of the GP across diagnostic and referral processes. Therefore, it was felt that considering other sources of referral information would be outside the scope. We have amended the third research objective to clarify this. We will also revisit this point in the discussion section of our scoping review paper. The third objective now includes the following text:

*What is the recommended referral process* *outlined in CPGs* *when referring a patient with suspected dementia from primary to secondary care?*

**Comment 4**

**Reviewer’s comment:**

 “In the methods section, it might be useful if the authors provide a definition for the word 'published’, e.g., available on GP professional organisations' websites (which can include national or international guidelines endorsed by professional organisations). As above, and in addition, given the 2023 SR by Monfared et al., it may be sufficient for the authors to review recommendations specific to general practice.”

**RESPONSE:** Thank you we appreciate that this needs clarifying. We have added some additional information to clarify what is meant by ‘published’. You make an interesting observation about the review by Monfared et al. (2023), and we did consider this. Although the authors reviewed screening and diagnosis recommendations, it was not within the scope of their review to consider the recommended role of the GP. In addition, many of the included CPGs in the Monfared et al. review were published in 2018, and we felt it important to gain more up-to-date data if possible.  We have now added the following text to the section ‘identifying relevant studies’:

*We will also search the websites of GP professional organisations nationally and internationally as guidelines are often made available for GPs through these platforms.*

**Comment 5**

**Reviewer’s comment:**

 “Identifying relevant studies – ‘ *we will also search the websites of GP professional organisations both nationally and internationally*’ Consideration will need to be given to access – if the guidelines are not open access will these be excluded or will the summary (which often is open access) be included. Will the authors contact the organisation directly to gain access to the required CPGs”. 

**RESPONSE:** Yes, thank you. We will contact the organisations to gain access, which has now been clarified in the method section. We do not intend to include open-access summaries, and we have now clarified this in the exclusion criteria. The following text indicates these changes:

*Where dementia guidelines are unavailable (or inaccessible) on the professional organisation websites, we will contact them via email to request a copy. Google Translate will be used to increase the likelihood of a response from international GP organisations.*

*(Method section: Identifying relevant studies)*

*Summaries of CPGs (added to exclusion criteria)*

**Comment 6**

**Reviewer’s comment:**

 “Inclusion criteria – *appropriate* for use in primary care – if not including general practice/primary care in the search terms, the word appropriate will need to be clearly defined. Exclusion – *guidelines for use in hospital settings* – suggest clarify if hospital setting and secondary care are synonymous“

**RESPONSE:** We appreciate that this may be unclear; therefore, we have removed the criteria ‘appropriate for use in primary care’. The next point ‘relevant for the diagnosis of dementia in primary care’ indicates our approach to inclusion and outlines what is meant by ‘relevant’. We have amended the exclusion criteria section to clarify what is meant by hospital settings.  The revised text reads:

*Guidelines for use in hospital in-patient settings.(Exclusion criteria)*

**Comment 7**

**Reviewer’s comment**

 “Guideline/guidance – the inclusion criteria is specific to 'guideline’ therefore suggest remove guidance or include an explanation for the word 'guidance' in the inclusion criteria”.

**RESPONSE**: Thank you.  For clarity, we have removed the word guidance.
